# Daily Cropland Soil NO_x_ Emissions Identified by TROPOMI and SMAP

**DOI:** 10.1029/2020GL089949

**Published:** 2020-11-13

**Authors:** Daniel E. Huber, Allison L. Steiner, Eric A. Kort

**Affiliations:** ^1^ Department of Climate and Space Sciences and Engineering University of Michigan Ann Arbor MI USA

## Abstract

We use TROPOMI (TROPOspheric Monitoring Instrument) tropospheric nitrogen dioxide (NO_2_) measurements to identify cropland soil nitrogen oxide (NO_x_ = NO + NO_2_) emissions at daily to seasonal scales in the U.S. Southern Mississippi River Valley. Evaluating 1.5 years of TROPOMI observations with a box model, we observe seasonality in local NO_x_ enhancements and estimate maximum cropland soil NO_x_ emissions (15–34 ng N m^−2^ s^−1^) early in growing season (May–June). We observe soil NO_x_ pulsing in response to daily decreases in volumetric soil moisture (VSM) as measured by the Soil Moisture Active Passive (SMAP) satellite. Daily NO_2_ enhancements reach up to 0.8 × 10^15^ molecules cm^−2^ 4–8 days after precipitation when VSM decreases to ~30%, reflecting emissions behavior distinct from previously defined soil NO_x_ pulse events. This demonstrates that TROPOMI NO_2_ observations, combined with observations of underlying process controls (e.g., soil moisture), can constrain soil NO_x_ processes from space.

## Key Points

- Daily TROPOMI data provide new opportunities to observe regional cropland NO_x_ emissions from space
- Soil NO_x_ pulsing is identified throughout the growing season with a NO_x_ maximum observed when soils dry to ~30% volumetric soil moisture
- Cropland NO_x_ emissions peak at the onset of the growing season as determined by TROPOMI NO_2_ enhancements and a box model framework

## 1 Introduction

Soils are a significant source of nitrogen oxides (NO_x_ = NO + NO_2_) to the atmosphere, contributing up to 40% of the nitrogen dioxide (NO_2_) column over cropland during Northern Hemisphere summer months (Hudman et al., 2012; Vinken et al., 2014). Fossil NO_x_ emissions, the largest source of NO_x_ in the troposphere, decreased on average by 5.9% year^−1^ from 2005–2017, increasing the relative contribution from soil NO_x_ to overall NO_x_ emissions (Jiang et al., 2018; Silvern et al., 2019). NO_x_ is a primary air pollutant associated with the formation of secondary pollutants including ozone (O_3_) and nitrogen‐based aerosols (Jenkin & Clemitshaw, 2000). NO_x_ and its subsequent oxidation products are not only detrimental to human health, but they can also cause adverse impacts for plants and other living organisms (Ashmore, 2005; Kampa & Castanas, 2008). As soil NO_x_ continues to represent a larger portion of total global NO_x_, it will be increasingly important to understand its emission on finer temporal and spatial scales.

Soil NO_x_ is primarily emitted in the form of nitric oxide (NO) with emissions driven by microbial processes within the soil surface layer (Pilegaard, 2013). The activity of NO‐producing bacteria is determined by environmental conditions such as water‐filled pore space (WFPS), soil temperature, and defining soil characteristics such as texture, bulk density, and nitrogen availability (Ludwig et al., 2001). WFPS plays a key role in controlling the magnitude of soil NO_x_ emissions, as the activity of bacteria that drive emissions is highly dependent on the ratio of water to oxygen in the soil pore space. The relative magnitude of soil NO_x_ emissions as a function of WFPS is typically represented by a Poisson function, with weakest emissions at extreme lower and upper limits of WFPS and strongest relative emissions between 20% and 65% WFPS (Hudman et al., 2012; Pilegaard, 2013), dependent upon specific soil characteristics. Increased nitrogen availability in cropland soils, largely due to fertilizer application, greatly enhances soil NO_x_ emissions (Bouwman et al., 2002; Oikawa et al., 2015), making croplands important sources contributing to the regional NO_x_ budget.

Current process understanding of soil NO_x_ emissions has been driven by small‐scale (~1 m) chamber studies, with emissions identified from a variety of soil and ecosystem types (e.g., Eberwein et al., 2020; Levine et al., 1996; Roelle et al., 2001; Schindlbacher et al., 2004). These and other observational studies of soil NO_x_ fluxes have been used to develop process‐based emissions models to estimate soil NO_x_ emissions, such as the Berkeley Dalhousie Soil NO_x_ Parameterization (BDSNP) (Hudman et al., 2012). BDSNP has been implemented into chemical transport models, including the GEOS‐Chem global model and CMAQ regional model (Hudman et al., 2012; Rasool et al., 2016). BDSNP represents the effects of environmental variables on the magnitude of emissions, including WFPS, soil temperature, soil nitrogen availability, soil biome, and the contribution to emissions from soil NO_x_ pulsing. NO_x_ pulsing refers to enhanced emissions that can occur after the first soil wetting following an extended dry period. The wetting of dry soil can reinvigorate previously dormant soil bacteria, resulting in NO emissions pulses that can be many times the prepulse emissions magnitude (Kim et al., 2012). The pulsing mechanism within the BDSNP is based on Yan et al. (2005), which activates once soils dry to a volumetric soil moisture (VSM) of 17.5% or less for at least three consecutive days prior to soil wetting.

Space‐based observations are particularly useful for understanding soil NO_x_ emissions in regions where ground‐based observations are not available. Using SCIAMACHY (Scanning Imaging Absorption spectroMeter for Atmospheric CHartographY) and a soil NO_x_ emissions model, Bertram et al. (2005) identified daily soil NO_x_ pulse emissions of up to 25 ng N m^−2^ s^−1^ in an agricultural region in Montana, with peak emissions at the beginning of the growing season. A global study used observed NO_2_ vertical column densities (VCDs) from the Ozone Monitoring Instrument (OMI) and the GEOS‐Chem model to quantify average June Northern Hemisphere soil NO_x_ emissions at 2.5° resolution (Vinken et al., 2014). Multiple satellite studies have observed soil NO_x_ emissions and pulsing in the African Sahel (Hickman et al., 2018; Jaeglé et al., 2004; Zörner et al., 2016), where NO_2_ column enhancements up to 100% of the prepulse VCDs are attributed to soil NO_x_ pulsing associated with the onset of the rainy season following months of dry weather (Zörner et al., 2016).

While satellite observations have been used to identify soil NO_x_ emissions in the past, no satellite study has yet constrained emissions at near‐daily regional scales and in conjunction with satellite‐observed process controls. In this study, we utilize satellite observations of tropospheric NO_2_ from TROPOMI (TROPOspheric Monitoring Instrument) to quantify the contribution of cropland soils to regional NO_x_ emissions in the lower Mississippi (MS) River Valley on daily to seasonal scales in 2018 and 2019. The unprecedented resolution of the TROPOMI product allows for soil emission processes to be evaluated using observed NO_2_ enhancements at spatiotemporal scales unresolvable with previous space‐based NO_2_ products. We identify a robust seasonally varying contribution from cropland soils to NO_x_ emissions, with the largest contributions during the late spring months (May–June), with emissions patterns matching predictions by the BDSNP model. Further, we use daily TROPOMI tropospheric NO_2_ observations in conjunction with Soil Moisture Active Passive (SMAP) VSM observations to identify NO_x_ pulse events in the days following precipitation, a consistently observed feature for this domain distinct from the historical definition of soil NO_x_ pulsing.

## 2 Data

Level 2 tropospheric NO_2_ VCD measurements are obtained from the TROPOMI instrument onboard the Sentinel‐5P satellite (Veefkind et al., 2012). TROPOMI was launched in 2017 and measures NO_2_ VCDs with a nadir spatial resolution of 3.5 × 7 km^2^ for observations between 30 April 2018 and 6 August 2019 and a resolution of 3.5 × 5.5 km^2^ from 6 August 2019 onward. TROPOMI uses observed radiation in the near‐ultraviolet and visible together with a chemical transport model to estimate tropospheric NO_2_ VCDs. We filter the TROPOMI data using only pixels with “flag_value” greater than or equal to 0.75 (van Geffen et al., 2019) to remove pixels that have unreliable measurements (e.g., due to the presence of clouds). To ensure that a sufficient number of pixels remain within the region of interest after applying this filter, we require that (1) a threshold of 30 pixels must remain within the domain after filtering based on the flag value alone and (2) the number of filtered pixels divided by the total number of pixels before filtering must be greater than or equal to 25%. If at least one of these conditions is not met, then the daily swath is excluded from analysis.

Level 3 surface VSM observations are obtained from the SMAP satellite (Entekhabi et al., 2010). SMAP was launched in 2015 and uses a passive microwave radiometer to observe surface radiation in the L‐band (1.4 GHz) to determine VSM mixing ratios in approximately the top 5 cm of soil. Measuring radiation at these wavelengths allows observations to be made in even very cloudy conditions, resulting in more temporally homogenous observations than TROPOMI NO_2_ observations, which are impacted by the presence of clouds. To ensure that SMAP VSM is measured from soils and not overlying vegetation, we apply a filter to remove pixels with vegetation water content greater than 5 kg m^−2^ (Colliander et al., 2017).

Daily winds are derived from ERA5 reanalysis (Hersbach et al., 2020) for 18:00–19:00 UTC, coincident with the TROPOMI overpass. Daily precipitation totals are from the NOAA CPC Gauge‐Based precipitation analysis (Chen et al., 2008). For the quantification of soil NO_x_ emissions, anthropogenic NO_x_ emissions are obtained from the 2014 gridded National Emissions Inventory (NEI) (Strum et al., 2017).

## 3 NO_2_ and Cropland in the Mississippi River Valley

**Figure 1:**
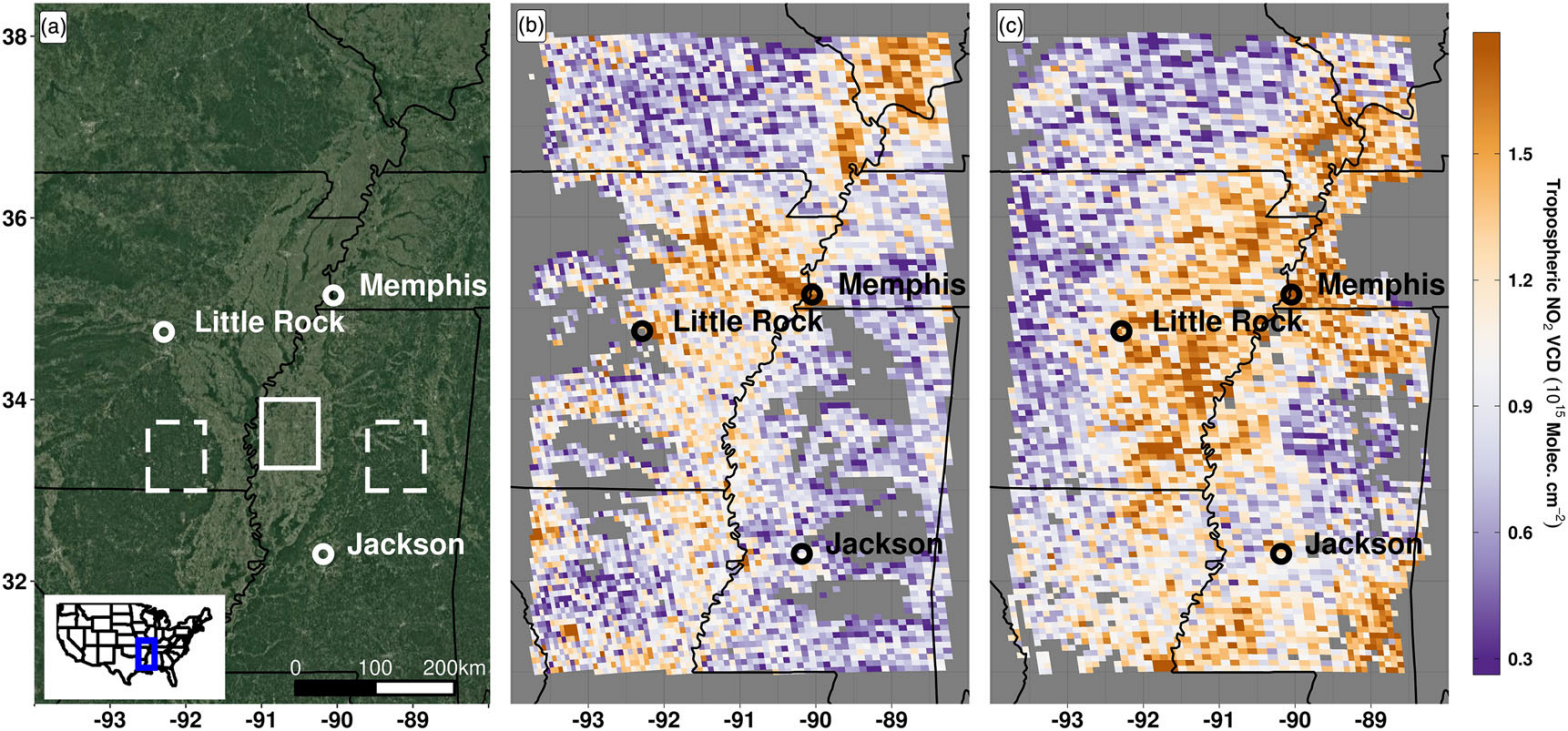
(a) Satellite image of the Mississippi Delta study region showing cropland (solid white box) and upwind (dashed white boxes) domains used to calculate daily NO_2_ enhancements. Light green regions are primarily cropland; dark green regions are primarily forest. (b) TROPOMI tropospheric NO_2_ VCDs on 14 May 2019, 5 days before a precipitation event. (c) TROPOMI tropospheric NO_2_ VCDs on 24 May 2019, 5 days after a precipitation event, with enhanced NO_2_ VCDs over cropland indicative of a drydown soil NO_x_ emissions pulse.

We define a 0.75 × 0.75° cropland domain located in the southern United States within the MS Delta (Figure 1a, solid white box). Soybean is the dominant crop type, representing nearly 80% of the cropland area as determined by the CropScape database (Han et al., 2012). This region experiences year‐round precipitation, with 28% and 19% of the annual precipitation occurring during the spring and summer seasons, respectively. This region regularly experiences changes in soil moisture due to rainfall as well as seasonal flooding from the MS River, which makes this an ideal location for studying the impact of soil moisture changes on soil NO_x_ emissions. Multiple power plants are located north of the study region that can substantially contribute to the local NO_x_ signature. Limiting our analysis to the MS Delta, which is located more than 125 km from the nearest major urban region or major power plants, greatly minimizes the influence of fossil NO_x_ emissions on the cropland NO_x_ signature. The cropland region has an east‐west extent of approximately 70 km and is adjacent to forest on both the eastern and western edges of the region.

Individual TROPOMI overpasses can spatially resolve increased NO_2_ VCDs over the cropland domain during drydown periods in days following precipitation (Figure 1). NO_2_ VCDs are relatively low over the cropland region 5 days before a rainfall event (Figure 1b; 14 May 2019) yet increase 5 days after (Figure 1c; 24 May 2019). Common features in the NO_2_ signal are evident on both days, including the anthropogenic NO_x_ signature from fuel combustion sources near Little Rock, AR, and Memphis, TN. However, the higher NO_2_ VCDs present over the cropland after the rainfall event (Figure 1c) suggest crop‐driven soil NO_x_ emissions in this region.

## 4 Results

### 4.1 NO_2_ Column Enhancement

We use forested regions upwind of the cropland domain as reference sites to estimate daily average background TROPOMI NO_2_ VCDs (Figure 1a, dashed white boxes). These reference sites provide nearby, “clean” upwind domains containing few major NO_x_ sources, which are ideal for background quantification. The reference sites facilitate the calculation of the NO_2_ column enhancement over the cropland domain by subtracting the average inflow background NO_2_ VCD from the average cropland NO_2_ VCD. This calculated difference reveals the contribution from cropland soils to the NO_2_ column for each day of available TROPOMI observations. A positive enhancement indicates higher cropland NO_2_ VCDs for that day, and a negative enhancement indicates higher upwind NO_2_ VCDs for that day. Upwind domains have been used in previous satellite studies to estimate background concentrations of atmospheric trace gases to derive enhancements (e.g., Kort et al., 2012) and offer a slight improvement over defining enhancements when using the lowest decile of observations (e.g., de Gouw et al., 2020). The high density of TROPOMI observations enables statistically robust daily evaluation of enhancements even considering the 30‐pixel requirement (see section 2) for both the upwind and cropland domains.

Depending upon the predominant wind direction, one of two different 0.75 × 0.75° upwind domains are defined for calculating the daily NO_2_ enhancement: one east and one west relative to the cropland domain (Figure 1a and supporting information Figure S1). Days with a predominantly northly wind (340–20°) are excluded due to the potential influence of urban emissions from the Memphis metropolitan region. Over the analysis period (2018–2019), the east domain is used for 37% of the daily enhancements, the west domain is utilized for 50% of the daily enhancements, and about 13% of days are excluded due to the presence of a predominantly northly wind.

### 4.2 Seasonal NO_2_ Enhancements and Soil NO_x_ Emissions Estimate

**Figure 2:**
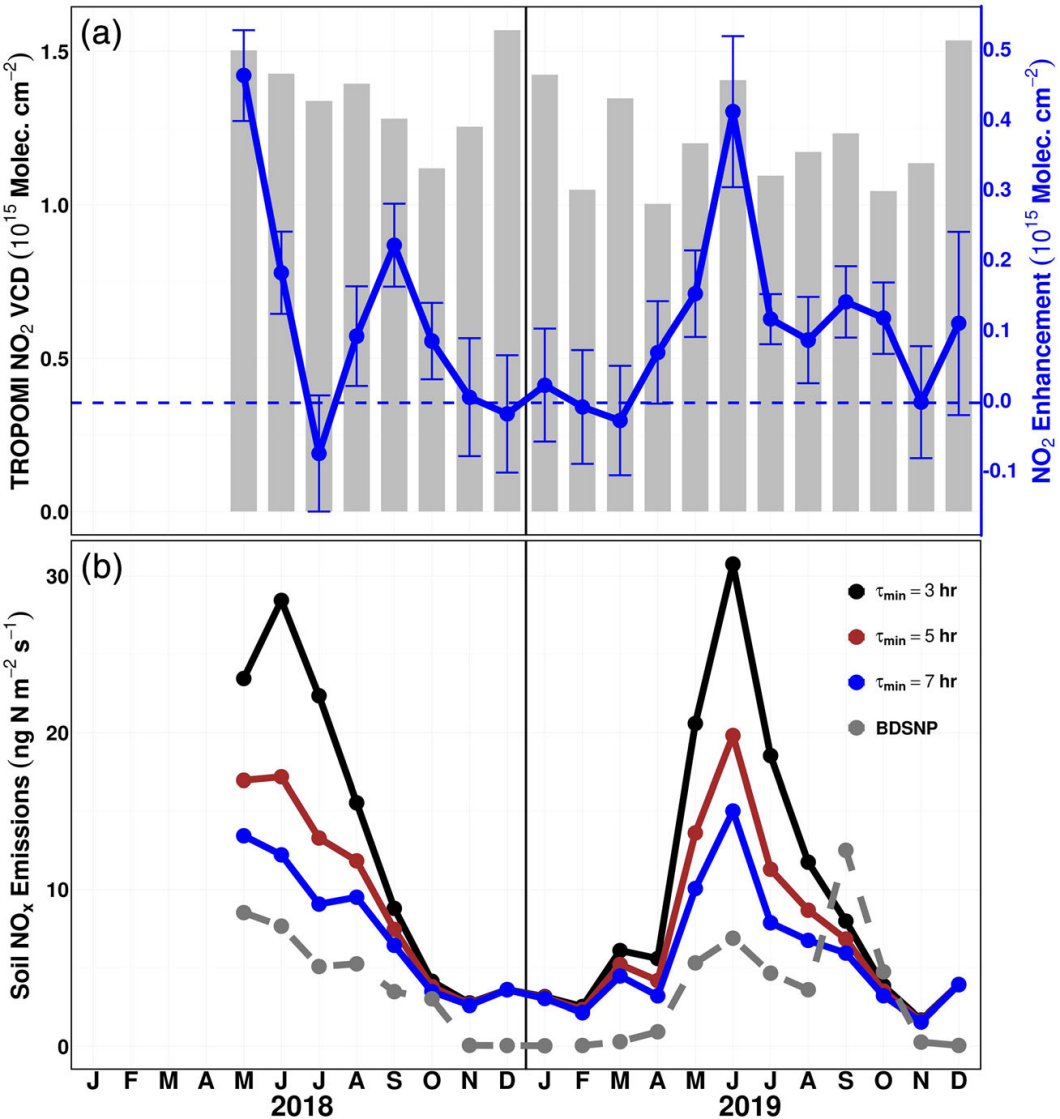
(a) Mean monthly TROPOMI tropospheric NO_2_ VCD (gray bars; left axis) and mean monthly NO_2_ column enhancement (blue line; right axis). NO_2_ enhancements represent the mean monthly contribution from cropland soils to the NO_2_ column. Error bars show standard error of the mean. (b) Monthly average soil NO_x_ emissions estimated using the soil NO_x_ emissions box model and the BDSNP model. All three box model scenarios converge to a December NO_x_ lifetime of 15 hr.

Monthly averaged NO_2_ enhancements are largest in May 2018 and June 2019 (Figure 2a), months that coincide with the onset of the growing season and an increase in agricultural activity. The average monthly enhancements during these months are between 0.4 and 0.5 × 10^15^ molecules cm^−2^. Enhancements in winter months are mostly negligible, coinciding with a relative lack of agricultural activity and resulting in similar NO_2_ VCDs over the cropland and upwind domains. Additionally, the timing of crop planting in the region largely shifts from May in 2018 to June in 2019 (USDA, 2019; Figure S2), suggesting that the shift in the largest TROPOMI enhancements from May in 2018 to June in 2019 is a direct result of the delayed planting of crops within the cropland domain. The magnitudes of these peak monthly enhancements are consistent with Vinken et al. (2014) that estimated an absolute contribution from soils to the NO_2_ column over cropland in the midwestern United States of approximately 0.6 × 10^15^ molecules cm^−2^ using the OMI satellite. However, Vinken et al. (2014) did not identify a contribution from soils to the NO_2_ column over the MS Delta cropland domain used within this study. This may be due to the coarser resolution of the model used (2.5°), coarser resolution of OMI relative to TROPOMI, or the higher fossil NO_x_ emissions during the study period (2005) that potentially masked the soil NO_x_ signal.

To estimate soil NO_x_ emissions (*E*_*soil*_: ng N m^−2^ s^−1^) from the cropland domain using TROPOMI NO_2_ observations, we apply a box model that accounts for sources and sinks of NO_x_:

(1) $$E_{\text{soil}}=\frac{U\;\Delta \left({\mathrm{NO}}_{2,\mathrm{VCD}}\right)}{L}+\frac{V_{d}\;{\mathrm{NO}}_{2,\mathrm{VCD}}}{Z_{\mathrm{PBL}}}+\frac{{\mathrm{NO}}_{2,\mathrm{VCD}}}{\tau }-E_{\mathrm{NEI}}$$

where the first term on the right‐hand side ($\frac{U\;\Delta \left({\mathrm{NO}}_{2,\mathrm{VCD}}\right)}{L}$) represents the advection of NO_x_ into the box, *U* is the average wind speed (m s^−1^) over the cropland domain, Δ(*NO*_2,*VCD*_) is the spatial TROPOMI NO_2_ column enhancement (molecule m^−2^) between the cropland and upwind domain, and *L* is the distance (m) from edge to edge between the cropland and the upwind domain (Figure 1a). The second term on the right‐hand side represents the deposition of NO_x_, where *V*_*d*_ is the NO_2_ deposition velocity (m s^−1^) from Yang et al. (2010), *NO*_2,*VCD*_ is the NO_2_ VCD (molecule m^−2^) over the cropland domain, and *Z*_*PBL*_ is the boundary layer height estimated at a constant 10^3^‐m height throughout the year. The third term on the right‐hand side represents the NO_x_ chemical loss rate, where $\frac{1}{\tau }$ is the inverse NO_x_ lifetime (s^−1^). The NO_x_ lifetime *τ* is estimated to vary sinusoidally throughout a year, with a peak lifetime on 21 December and a minimum lifetime on 21 June. *E*_*NEI*_ is the anthropogenic NO_x_ emissions (molecule m^−2^ s^−1^) from the 2014 NEI inventory. Chemical production as a source of NO_x_ is assumed to be negligible.

Using Equation 1, we calculate daily box model estimates of soil NO_x_ emissions and average to monthly values (Figure 2b). We present three different emissions scenarios with a varying minimum June NO_x_ lifetime of 3, 5, and 7 hr. Martin et al. (2003) estimated NO_x_ lifetime of approximately 5 hr in the summer at this latitude, and we increase and decrease the summer lifetime by ±2 hr to illustrate the sensitivity of the box model emissions estimates to NO_x_ lifetime assumptions. All three scenarios converge to a maximum December NO_x_ lifetime of 15 hr. The largest emissions occur during the late spring and early summer, with minimal emissions during the winter. Our monthly average box model emissions estimates range from 15 to 34 ng N m^−2^ s^−1^ in May and June, with the range driven by the influence of NO_x_ lifetime as described in recent studies (e.g., Laughner & Cohen, 2019; Shah et al., 2020).

For the same domain and time frame, we estimate emissions using the BDSNP model. As inputs for the BDSNP, we use WFPS calculated from SMAP surface VSM observations, ERA5 soil temperature (Hersbach et al., 2020), and soil nitrogen availability data available from the MEGAN biogenic emissions model framework (Guenther et al., 2006). WFPS is calculated from the ratio of SMAP VSM to estimated soil porosity within the cropland domain (Linn & Doran, 1984). BDSNP soil NO_x_ emission magnitudes are roughly half that of the box model emissions with a 5‐hr June lifetime; however, the month‐to‐month variability between the two methods is consistent (Figure 2b). Both methods estimate relative peak emissions in May of 2018 and June of 2019. Further, both methods experience similar month‐to‐month variability during the growing season. The exception to this is September of 2019, during which BDSNP estimates the largest monthly average emissions for the entire study period.

Our satellite‐based soil NO_x_ emission estimates are largely consistent with small‐scale chamber studies as well as satellite studies. A chamber study over cropland in North Carolina, United States, measured average NO emissions on the order of 20.2 ± 19 ng N m^−2^ s^−1^ during spring and summer (Roelle et al., 2001), while a chamber study in high‐temperature croplands in Southern California observed median emissions of 20 ng N m^−2^ s^−1^ with individual measurements up to 900 ng N m^−2^ s^−1^ (Oikawa et al., 2015). Satellite studies show similar ranges, with Bertram et al. (2005) using SCIAMACHY to estimate May soil NO_x_ emissions from cropland in Montana, United States, with daily values ranging from 10 to 25 ng N m^−2^ s^−1^ and Jaeglé et al. (2004) used the Global Ozone Monitoring Experiment (GOME) instrument to estimate average June soil NO_x_ emissions from the Sahel region of 20 ng N m^−2^ s^−1^ under the assumption of a 7‐hr NO_x_ lifetime.

### 4.3 Daily‐Scale NO_2_ Enhancements and Multiday NO_x_ Pulse Events

To observe the relationship between soil emissions and soil moisture within the cropland domain, we use SMAP VSM observations to identify soil drydown events that occur in the days following precipitation and observe changes in daily TROPOMI NO_2_ enhancements during those events. We identify days in 2018 and 2019 between May and October with heavy (≥1 cm) precipitation followed by at least 1 week without heavy precipitation. We require observed VSM to increase to greater than 0.4 cm^3^ cm^−3^ in response to the initial precipitation and then decrease in the week following without a subsequent increase. If a relative peak in TROPOMI NO_2_ enhancements occurs as SMAP observations decrease in the week following precipitation, then the peak enhancement is associated with a “drydown NO_x_ pulse” event.

**Figure 3:**
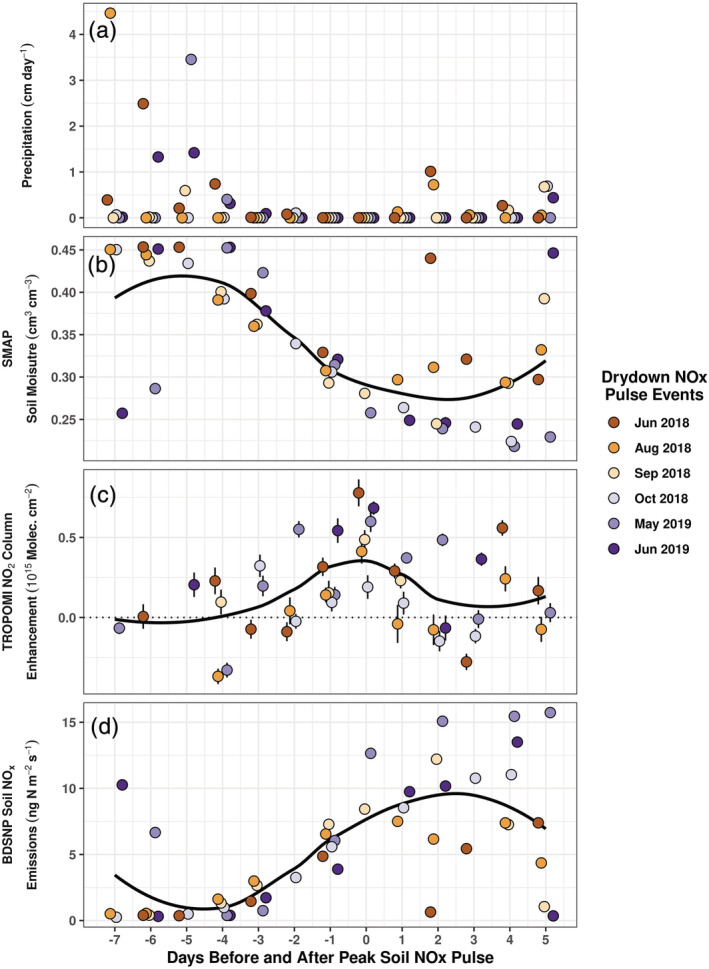
Time series for six drydown NO_x_ pulse events showing (a) NOAA CPC unified gauge‐based precipitation, (b) observed SMAP VSM, (c) observed TROPOMI NO_2_ column enhancements, and (d) estimated BDSNP soil NO_x_ emissions. Data points for each day are slightly offset to aid visibility. Black curves represent smoothed local regression. Vertical standard error bars are included for SMAP and TROPOMI observations (panels b and c). Error bars in panel b are small and are covered by the markers. NO_2_ column enhancements are defined as the VCD difference between the cropland box and the upwind box (Figure 1a). Day 0 is defined as the day on which the peak drydown NO_x_ pulse occurs following an observed decrease in SMAP observations to ~30% VSM.

Using the above criteria, we identify nine potential drydown NO_x_ pulse events between May and October in 2018 and 2019. Two drydown events are excluded due to the absence of TROPOMI data. One event is excluded due to persistently high TROPOMI enhancements occurring before, during, and after soil drying. We align the remaining six events onto the same day axis, defining Day 0 as the day of relative peak NO_2_ enhancement following the decrease in soil moisture (Figure 3). NO_2_ enhancements increase as the soil dries and enhancements reach a relative maximum on Day 0, coincident with VSM decreasing below a value of 0.3 cm^3^ cm^−3^. This suggests a local SMAP VSM threshold of approximately 30%, an emergent observation below which soils must decrease for drydown pulse emissions to reach a maximum. A previous study has shown that SMAP observations may exhibit faster soil drying than in situ measurements (Shellito et al., 2016), which would suggest that the observed threshold may be offset from in situ measured soil moisture. Notably, in a chamber study on cropland NO emissions in California, Oikawa et al. (2015) found that peak soil NO_x_ emissions occurred at roughly 30% VSM, suggesting that this 30% threshold may hold broader significance for cropland soils. We evaluate the significance of each Day 0 NO_2_ column enhancement by conducting two‐sample *t* tests between upwind and cropland domain observations for all six events, confirming the significance of the observed enhancements (*p* values < 0.05 for five of six events, *p* value = 0.09 for remaining event).

The drydown NO_x_ pulsing we observe is distinct from NO_x_ pulsing as classically described in the literature. Soil NO_x_ pulsing is historically characterized by a substantial increase in soil NO emissions within hours after soil wetting following an antecedent dry period (Davidson, 1992; Kim et al., 2012). Here, we observe peak enhancements between 4 and 8 days after precipitation and in the absence of preceding dry periods (Figure 3). A multiday lag between soil wetting and peak soil NO_x_ pulse emissions is not unprecedented and is hypothesized in Hall et al. (1996). A lag of 2–7 days has been observed (Hickman et al., 2018; McCalley & Sparks, 2008); however, both studies experience preceding dry conditions, a distinct difference from our findings.

We include BDSNP soil NO_x_ emissions estimates for the same period as the drydown pulse events to compare with the behavior in the observed NO_2_ column enhancements (Figure 3d). While BDSNP emissions increase following precipitation, emissions continue to increase even after the observed TROPOMI enhancements peak on day 0. This is a result of the modeled soil moisture dependency within the BDSNP which is designed to peak at 13% VSM (30% WFPS) in the cropland domain, causing BDSNP estimates to continue increasing as soils continue drying after Day 0. This may explain the largest BDSNP emissions during September 2019 (Figure 2), as that month was the only time during the study period during which VSM values approached, but did not reach, 13% for multiple days, causing the BDSNP to estimate greater emissions during that month. This implies that for some cropland soils, BDSNP may overestimate emissions at lower VSM, may underestimate emissions at higher VSM, and may not capture the pulsing during drydown periods identified in the satellite record.

## 5 Conclusions

We find that daily spatial TROPOMI NO_2_ enhancements can be successfully used to quantify the contribution from cropland soil to the NO_2_ column at the daily and the seasonal scales and can sufficiently resolve the spatial variability associated with soil NO_x_ emissions. The resolution of the TROPOMI NO_2_ product provides a much higher density of observations compared to previous satellite products, allowing for soil NO_x_ emissions to be resolved in spatially confined regions like the MS Delta. We show that daily TROPOMI NO_2_ observations can be applied to a box model framework to quantify seasonal cropland soil NO_x_ emissions for 2018 and 2019. Monthly NO_2_ enhancements peak in late spring and early summer, times at which agricultural activity increases and enhances cropland soil NO_x_ emissions. Peak monthly NO_2_ enhancements shift from May in 2018 to June in 2019, a shift that coincides with a shift in the timing of crop planting between 2018 and 2019. This suggests that seasonal land management practices directly influence the contribution from cropland soils to the NO_2_ column. Soil NO_x_ box model emissions estimates achieve an annual maximum ranging from 15 to 34 ng N m^−2^ s^−1^, values that are within the range of other estimated soil NO_x_ emissions. Box model emissions estimates are higher than BDSNP estimates, with the box model exhibiting similar variability in annual soil NO_x_ emissions as predicted by the BDSNP model. The lower BDSNP estimates may arise as a consequence of not capturing emissions that peak at VSM values above 13% in the cropland domain.

Additionally, TROPOMI NO_2_ enhancements can resolve drydown NO_x_ pulse emissions over cropland in conjunction with decreasing SMAP surface VSM observations in the days following precipitation. This highlights a unique application of two space‐based instruments to observe daily environmental process controls that contribute to enhanced cropland NO_x_ emissions. The daily soil contribution to the NO_2_ column during peak drydown NO_x_ pulsing ranges from 0.2 × 10^15^ molecules cm^−2^ (October 2018) to 0.8 × 10^15^ molecules cm^−2^ (June 2018), consistent with more abundant available soil nitrogen at the beginning of the growing season (May/June) and less abundant at the end of the growing season (October). During drydown NO_x_ pulsing, TROPOMI NO_2_ enhancements peak in the week following precipitation once SMAP measurements decrease below a threshold of 30% VSM (65% WFPS). This implies that not all nonarid soils experience peak emissions at 30% WFPS as is currently implemented in the BDSNP and that BDSNP emissions may be underestimated or overestimated in regions where different soil moisture responses exist.

## Supporting information

Supporting Information S1

## Data Availability

Data used in this paper are downloaded from the Sentinel‐5P Pre‐Operations Data Hub (TROPOMI, https://s5phub.copernicus.eu/dhus/), the National Snow and Ice Data Center (SMAP, https://nsidc.org/data/SPL3SMP_E/versions/3), and the ECMWF Climate Data Store (ERA winds, https://cds.climate.copernicus.eu/cdsapp#!/dataset/reanalysis‐era5‐single‐levels?tab=overview).
