# Supplementary material for: Daily Cropland Soil NOx Emissions Identified by TROPOMI and SMAP
Source: Geophys Res Lett. 2020 Nov 13;47(22):e2020GL089949. doi: 10.1029/2020GL089949 (PMC7757188; doi:10.1029/2020GL089949)
Supplement: Supplementary file 1 — Supporting Information S1 [file GRL-47-e2020GL089949-s001.pdf]

AGU PUBLICATIONS

Geophysical Research Letters

Supporting Information for

Daily Cropland Soil NOx Emissions Identified by TROPOMI and SMAP

Daniel E. Huber, Allison L. Steiner, Eric A. Kort

Department of Climate and Space Sciences and Engineering, University of Michigan, Ann Arbor, MI

Contents of this file

Figures S1 to S2

Introduction

This supporting information provides additional context regarding the upwind domain selection for our analysis (Figure S1) and information regarding the timing of crop planting within the cropland study region for 2018 and 2019 (Figure S2).

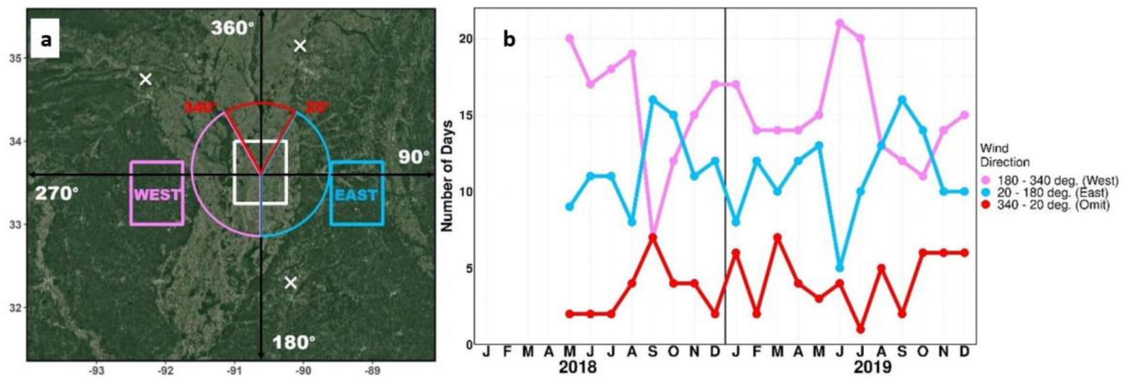

Figure S1. (a) Directional representation of upwind domain selection based on daily wind fields. The western (pink) domain is used on days with wind direction from 180° to 340°, and the eastern (blue) domain is used on days with wind direction from 20° to 180°. Days with northerly winds between 340° and 20° are excluded from analysis, due to the presence of powerplants north of the region near Memphis, TN. White x's represent major cities. (b) Number of days per month with either easterly or westerly winds, and number of omitted days with northerly wind direction.

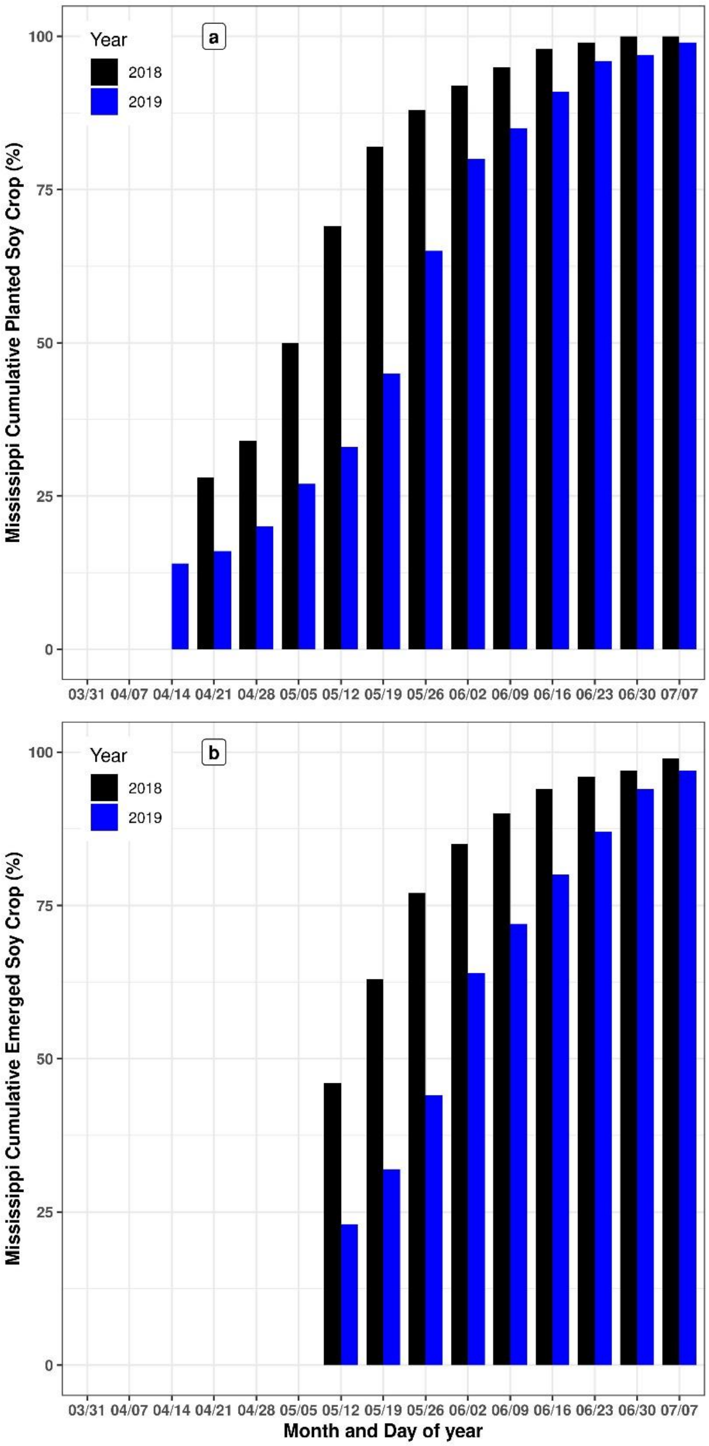

Figure S2. USDA soy crop progress reports for the state of Mississippi for 2018 (black) and 2019 (blue). (a) Cumulative soy crop planting by date. (b) Cumulative soy crop emergence by date. The shift in peak TROPOMI NO_2 enhancement from May of 2018 to June of 2019 (Fig. 3) may be explained by the shift in crop planting / emergence between 2018 and 2019.
